# Exhaled Aerosol Transmission of Pandemic and Seasonal H1N1 Influenza Viruses in the Ferret

**DOI:** 10.1371/journal.pone.0033118

**Published:** 2012-04-03

**Authors:** Frederick Koster, Kristine Gouveia, Yue Zhou, Kristin Lowery, Robert Russell, Heather MacInnes, Zemmie Pollock, R. Colby Layton, Jennifer Cromwell, Denise Toleno, John Pyle, Michael Zubelewicz, Kevin Harrod, Rangarajan Sampath, Steven Hofstadler, Peng Gao, Yushi Liu, Yung-Sung Cheng

**Affiliations:** 1 Program in Applied Science, Lovelace Respiratory Research Institute, Albuquerque, New Mexico, United States of America; 2 Program in Infectious Diseases, Lovelace Respiratory Research Institute, Albuquerque, New Mexico, United States of America; 3 Program in Aerosol Science, Lovelace Respiratory Research Institute, Albuquerque, New Mexico, United States of America; 4 Analytical Services Laboratory, Ibis Biosciences, Carlsbad, California, United States of America; 5 Program in Lung Cancer, Lovelace Respiratory Research Institute, Albuquerque, New Mexico, United States of America; The University of Hong Kong, China

## Abstract

Person-to-person transmission of influenza viruses occurs by contact (direct and fomites) and non-contact (droplet and small particle aerosol) routes, but the quantitative dynamics and relative contributions of these routes are incompletely understood. The transmissibility of influenza strains estimated from secondary attack rates in closed human populations is confounded by large variations in population susceptibilities. An experimental method to phenotype strains for transmissibility in an animal model could provide relative efficiencies of transmission. We developed an experimental method to detect exhaled viral aerosol transmission between unanesthetized infected and susceptible ferrets, measured aerosol particle size and number, and quantified the viral genomic RNA in the exhaled aerosol. During brief 3-hour exposures to exhaled viral aerosols in airflow-controlled chambers, three strains of pandemic 2009 H1N1 strains were frequently transmitted to susceptible ferrets. In contrast one seasonal H1N1 strain was not transmitted in spite of higher levels of viral RNA in the exhaled aerosol. Among three pandemic strains, the two strains causing weight loss and illness in the intranasally infected ‘donor’ ferrets were transmitted less efficiently from the donor than the strain causing no detectable illness, suggesting that the mucosal inflammatory response may attenuate viable exhaled virus. Although exhaled viral RNA remained constant, transmission efficiency diminished from day 1 to day 5 after donor infection. Thus, aerosol transmission between ferrets may be dependent on at least four characteristics of virus-host relationships including the level of exhaled virus, infectious particle size, mucosal inflammation, and viral replication efficiency in susceptible mucosa.

## Introduction

Seasonal influenza virus is a highly contagious respiratory pathogen that causes over one million infections and is associated with approximately 3000 up to 49,000 deaths each year in the United States [1]. Infections due to the 2009 H1N1 pandemic influenza virus were primarily self-limited with the highest attack rates among children and young adults [2]. Future pandemics could theoretically duplicate the high virulence of the 1918 pandemic [3], [4]. In addition to pre-pandemic vaccination, non-pharmaceutical interventions such as social distancing may have important roles in reducing transmission. The current poor quantitative understanding of influenza transmission could impair the development of evidence-based interventions [5], [6].

Transmission is classified as three modes, by direct contact with infected persons or indirect contact with contaminated fomites, by inhaling large droplets (droplet spray) within a meter of the source, or by small particle aerosols exhaled during talking, coughing or breathing [7], [8]. Reviews of influenza transmission among human populations have concluded that transmission is by contact and droplet spray [9], [10] while others conclude that aerosol transmission contributes significantly to spread of infection [11], [12]. Transmissibility is currently estimated from the spread of influenza in relatively closed populations and is expressed as the number of secondary infections derived from one contagious person (basic reproductive number R_0_). The R_0_ for the 2009 H1N1 pandemic strain has been estimated to range from 1.4 to 1.8 [13]–[16], values that overlap with the R_0_ of seasonal strains of 1.7–2.1 [17] and 1.3–4.9 for the 1918 pandemic [18]. The epidemiological R_0_, however, is complicated by unmeasured human variables including contact behavior and pre-existing immunity [19].

A quantitative measure of strain transmissibility independent of human variables would be useful in predicting epidemic potential. Transmissibility in an animal model is the combination of four serial components, first a threshold viral load in the respiratory mucosa of the contagious host, then exhalation of virus, survival of airborne virus, and finally replication kinetics of inhaled virus in susceptible mucosa [20]. Ferrets have served as a transmission model mimicking many features of human transmission [21], [22]. Key viral genotypic features in the hemagglutinin and PB2 segments related to relative transmissibility have been described in multiple subtypes [23]–[27]. Studies of non-contact droplet (aerosol) transmission between ferrets have utilized continuous exposures in a side-by-side cage design to identify relative differences in transmissibility between strains. Under these conditions H5N1 avian-origin viruses were not transmitted [28], whereas seasonal strains were readily transmitted, and pandemic strains were transmitted as readily [29] or less readily [30] than seasonal strains. Recently, the aerosol ID_50_ has been measured for two seasonal influenza strains in the ferret model [31], [32].

Our goals in this study included measuring the viral load in the exhaled aerosol under controlled airflow conditions and exposing susceptible ferrets to exhaled aerosols for only 3 hours to approximate the duration of typical human exposures. We examined ferret-to-ferret exhaled viral aerosol transmission for one seasonal and three pandemic H1N1 strains. Ferrets infected with the seasonal virus exhaled higher levels of virus than those infected with the pandemic strains, yet transmission of the pandemic strains was significantly more efficient than the seasonal strain.

## Methods

### Virus

Three strains of swine-origin pandemic 2009 H1N1 viruses A/California/04/2009 (Cal/04), A/Mexico/4482/2009 (Mex/4482) and A/California/07/2009 (Cal/07), and one seasonal H1N1 strain A/New Caledonia/20/99 (NC/99) were plaque-purified stocks obtained from the Influenza Division, Centers for Disease Control, with a history of two passages in embryonated eggs, and two passages in MDCK cells. Working stocks were prepared from two passages in embryonated eggs, aliquoted, and stored at −80°C up to four months until needed. The 2009 H1N1 viruses (Cal/04:FJ966079; Cal/07:FJ984387; Mex/4482:CY098505) were sequenced after passage to assure preservation of the consensus sequences according to the NIH Influenza Database.

Virus inocula were titered before inoculation by the focus neutralization assay (FFU) and embryonated egg infection (EID_50_ or 50% egg infectious dose) and after each inoculation for FFU. Aliquots were thawed at 4°C and diluted in PBS to 10^6^ FFU/mL for ferret inoculation. The titer of 10^6^ FFU was approximately equivalent to 1.2–1×10^6^ EID_50_ depending on the experiment. Donor ferrets were inoculated intranasally (i.n.) with 10^6^ FFU/mL, 0.5 mL in each naris. In one experiment donor ferrets were inoculated by both the i.n. and intratracheal (i.t.) routes with a combined total dose of 10^6^ FFU/mL. In one experiment donor ferrets were infected by nebulized virus as previously described [32]. Estimated delivered dose was calculated from viral RNA measured in the nebulized aerosol as described in the text.

### Animals

Outbred castrated male ferrets (Mustela putorius furo) were obtained from Triple F Farms (Sayre, PA) at 12−18 weeks old, were quarantined for 14 days and weighed 1.0–1.2 kg when exposed to the viruses. Ferrets were uniquely identified by ear tags and subcutaneous transponders (IPTT-300; Bio Medic Data Systems Inc, Seaford, Delaware). Ferrets were pair- or triple-housed in plastic cages with perforated plastic bottoms (Model RB 272718UP6 Rabbit cage modified to hold ferrets; Allentown Cage Inc, Allentown, NJ) during the quarantine period but singly housed after viral exposure in ventilated cages to prevent cross infection by isolating the air entering and leaving each cage. Excreta pans under the cages, cage flooring, and room floors were cleaned daily. All procedures were conducted under protocols approved by the Institutional Animal Care and Use Committee (IACUC) at Lovelace Respiratory Research Institute (permit #08-011), all facilities were accredited by the Association for Assessment and Accreditation of Laboratory Animal Care International (AAALAC). Guidelines for ferret housing and environment described in the Guide for the Care and Use of Laboratory Animals, Seventh Edition, National Research Council, were strictly adhered to.

### Aerosol Exposure Apparatus

**Figure 1:**
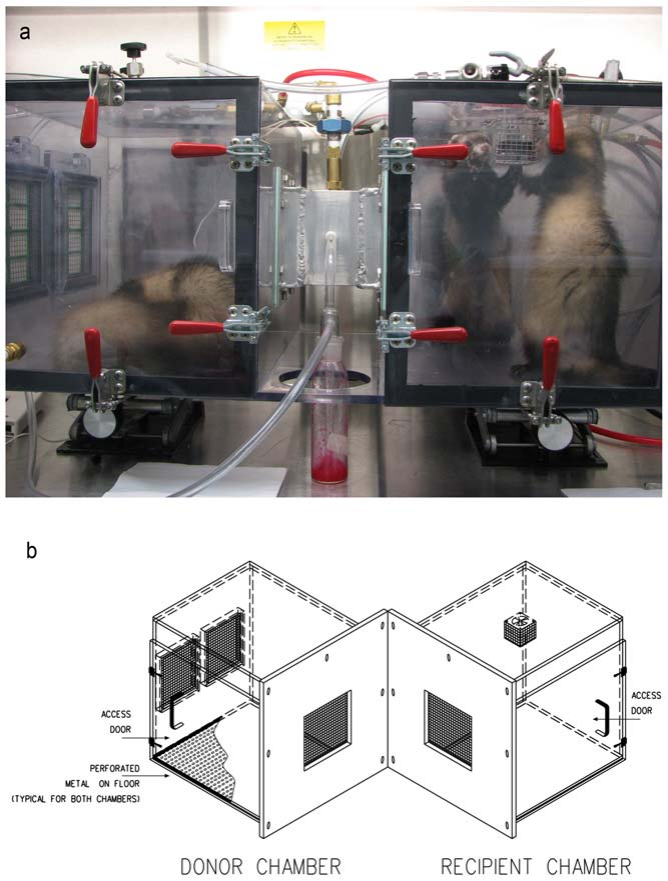
Exposure chambers. A . Photograph of tunnel exposure chamber occupied by two sleeping ferrets in the left donor chamber and two active ferrets in the right ‘recipient’ chamber. Note that experiments are described with two ferrets in the donor chamber but no experiments were performed with two ferrets in the recipient chamber. A line diagram of this exposure chamber appears in reference 32. Air is drawn through HEPA filters in the left wall of the donor chamber (left side of photograph) passes through the tunnel where particle size and PTFE filter ports are located, and is withdrawn through the recipient chamber (right side) and exits through two ports into HEPA filters (not visible in photograph). **B**. Exploded view of exposure chamber without tunnel between donor and recipient ferret, designed to more closely approximate conditions of side-by-side cage exposures in published ferret-model aerosol transmission studies.

Two exposure apparatuses were fabricated each consisting of a donor (infected ferret) chamber and a recipient (susceptible ferret) chamber with wire screens between the chambers to prevent direct contact (**Figure 1A, B**). The slightly smaller apparatus had a 7×7×15 cm long tunnel between the chambers and the larger apparatus had a screened 2 cm passage between the chambers. Air-flow was left-to-right, drawn into the left (donor) chamber at 12 L/min by controlled vacuum. In the tunnel or space between the donor and recipient chambers, three air sampling ports for polytetrafluoroethylene (PTFE) filters (Teflon, 37-mm, 2-m pore-size, Pall Life Sciences, NY) each withdrew 2 L/min, and a fourth port drew 1 L/min into a laser particle counter (Grimm). Air at 6 L/min entered the right (recipient ferret) chamber, was mixed by a fan and was removed through two HEPA filters in the right-side wall. Humidity (RH) and temperature were controlled by ambient laboratory air at approximately 45% (range 41–48) RH and 22°C respectively. After exposure, filters were put in 1ml serum-free media and vigorously vortexed for 20 seconds, the filter removed, a 500µl aliquot placed into 500µl of RNABee (Amsbio, Lake Forest, CA)and frozen at −80°C for RNA extraction, and the remaining fluid was frozen at −80°C for viral culture. After each exposure the chambers were washed with 1% bleach solution followed by repeated isopropyl alcohol rinses to remove all traces of viable virus and viral RNA. For the experiments infecting donor ferrets by nebulized virus, a six-jet Collison generator was loaded with 20 mL Eagles Minimal Essential Media and antifoam (Sigma) as previously described [32].

### Clinical Observations

Following aerosol challenge, ferrets were observed twice daily for symptoms including sneezing, cough, nasal discharge, respiratory distress, reduction in activity, neurologic signs, or abnormal behavior. Activity was scored using standard criteria (0 = alert and playful, 1 = alert but playful only when stimulated, 2 = alert but not playful when stimulated, and 3 = neither alert nor playful when stimulated). Temperature was measured by transponder from two subcutaneous microchips (IPTT-300 Implantable Programmable Temperature and Identification Transponder; Bio Medic Data Systems, Inc, (BMDS) Seaford, DE 19973) implanted over the shoulder and hip regions and recorded by BMDS electronic proximity reader wand (WRS-6007; BMDS) as the average of the two readings twice daily.

### Pathology

Ferrets were necropsied at protocol-determined intervals after exposure in order to image the gross lung pathology, measure viral load in upper respiratory tract, trachea and lung tissue, and assess the histological extent of infection. Lung lobe samples, trachea, nasal turbinates, liver, spleen, tracheobronchial lymph nodes, brain, and olfactory bulbs were processed for viral load and histopathology; results of extrapulmonary tissues will be reported elsewhere. Tissues for histopathology were fixed in 4% paraformaldehyde, embedded in paraffin, sectioned at 4 to 6 µm, stained with hematoxylin and eosin, and read by a veterinary pathologist without knowledge of the infection history. Selected lung tissue was fixed in methanol prior to incubation with anti-nucleoprotein antibody (Clontech) to correlate location of influenza antigen with histological characteristics of epithelial cell infection.

### Viral Load

Sample collection, viral culture, RNA isolation, RT-qPCR and T5000™ assays were performed as previously described [32]. Viral load was determined by standard tissue culture on MDCK cells reported as focus-forming units (FFU) and also by real-time quantitative RT-qPCR. For aerosol samples collected from PTFE filters, viral genomic RNA was measured by two PCR-based assays, a standard RT-qPCR detecting nucleocapsid gene RNA (Lovelace Respiratory Research Institute [LRRI]), and a more sensitive experimental RT-PCR-based system using time-of-flight mass spectroscopy analysis of amplimers to detect six influenza A segments (**T**5000^TM^ Biosensor System, Ibis Biosciences, Abbott Molecular) [33], [34]. Since recipient nasal washes were not cultured daily, but recipients had persistently positive viral RNA assays in daily throat swabs, infection was defined either by positive culture or by positive viral RNA levels >10^7^ genome equivalents (Geq) for three or more consecutive days [32]. Data on viral RNA in aerosol samples are reported only for the more sensitive T5000 assay unless noted as a comparison of the two assays.

### Calculation of Inhaled Virus

The viral RNA collected from the aerosol and analyzed by the T5000 assay was used to calculate the infectious virus dose inhaled by the exposed recipient ferret according to the formula:

Virus inhaled = (Geq vRNA/h)*(FFU/Geq ratio)*(MV/filter sample flow rate).

The FFU/Geq ratio was derived from 4 Teflon filter collections of high levels of aerosolized virus with positive culture and viral RNA data. The fraction of air inhaled was calculated from the minute ventilation (MV) divided by the airflow/min entering the recipient chamber (6 L/min). Minute ventilation was estimated from unpublished data and literature reports, adjusted for ferret weight and sedation status. For anesthetized ferrets weighing 280 g and 560 g, tidal volumes (TV) have been reported as 4.0 and 6.0 mL, respectively [35], [36]. TV for our ferrets weighing between 1.0 and 1.2 kg was calculated as 9.0 mL according to the ¾-power relationship between body weight and lung volume. The breathing rate (RR) was observed to be a mean of 35/min (range 28–43), and the lack of sedation was adjusted by multiplying the TV by 1.5 (J Mauderly, personal communication), yielding a calculated MV (TV*RR*1.5) of 472 mL/min.

The aerosol ID_50_ was estimated using Proc Probit in SAS 9.1 (Cary, NC) employing data from Figure 7 on calculated inhaled virus and whether or not the recipient ferret was infected according to criteria described in Methods, viral load.

## Results

### Aerosol Characteristics

**Figure 2:**
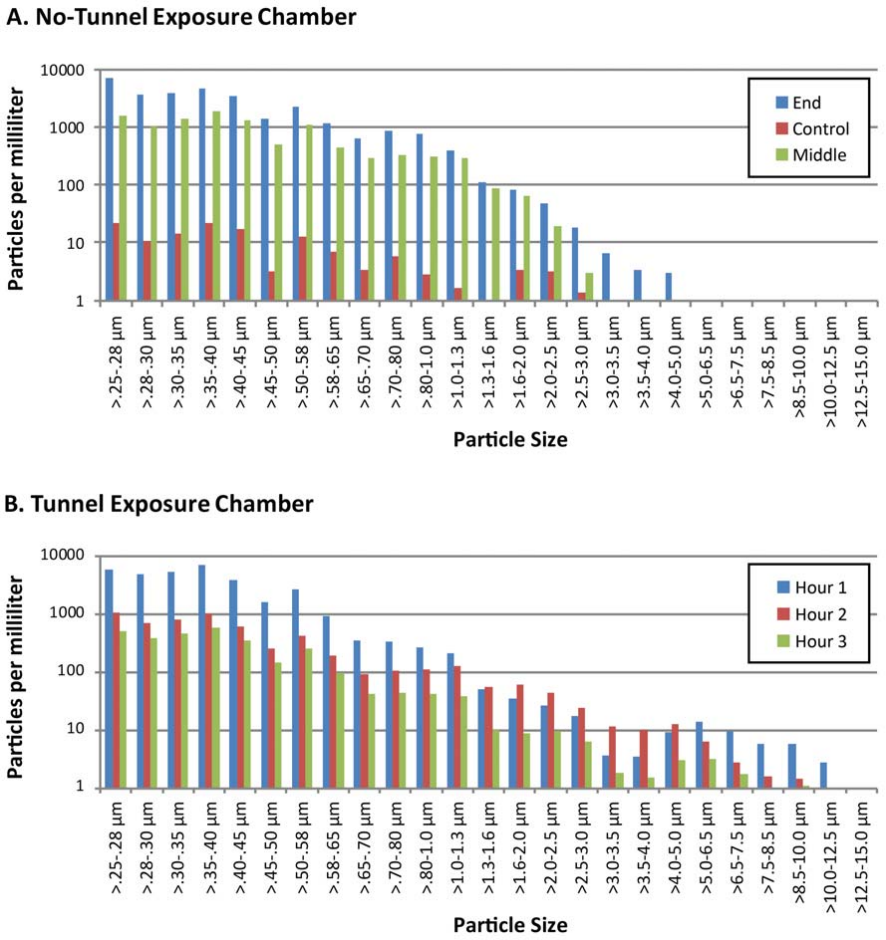
Distribution of particle dimensions delivered to recipient chamber. Particle diameter measured by laser light scattering plotted as log_10_ particles per liter of air divided into 24 diameter cohorts ranging from 0.25 microns to 12.5 microns. Particles sampled during 10 min. intervals with sampling airflow at 1.0 Lt/min and with one resting ferret infected with Cal/04 virus in donor chamber. Top graph: In side-by-side chamber sampling collected during the middle (green) and end (blue) of the same exposure period, and during an interval of no directed airflow when vacuum is off (control, red) particle numbers of all size cohorts decreased more than 100-fold. Bottom graph: In tunnel exposure chamber samples collected during first hour (blue), second hour (red) and third hour (green) of continuous 3 hour exposure.

**Figure 3:**
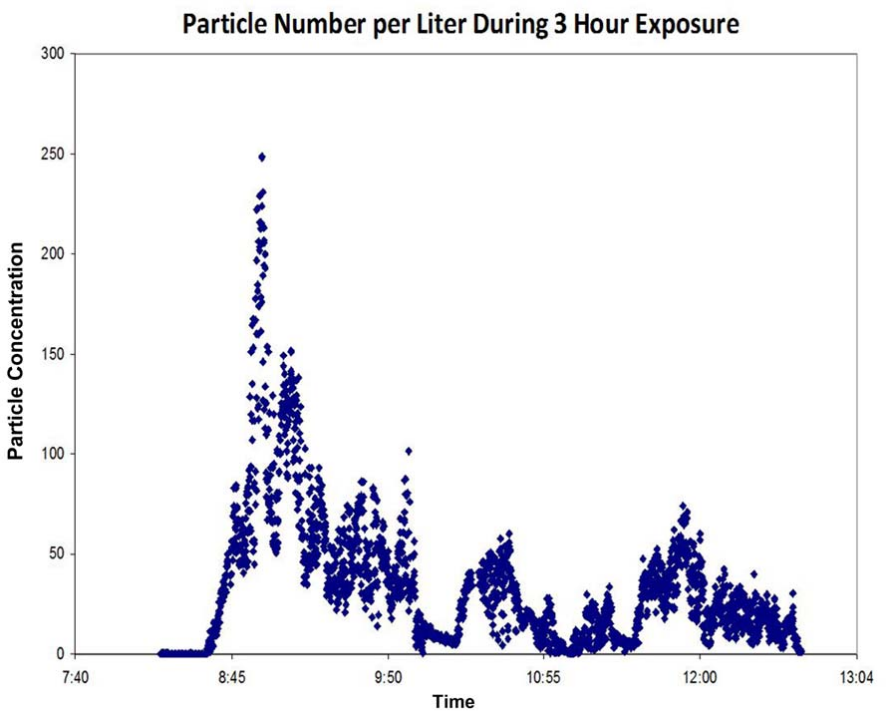
Particle Concentration variation during chamber exposure. Airborne particles were measured continuously by Grimm spectrometer and recorded as particles per liter of air in this typical exposure. Particle numbers per second vary over two orders of magnitude.

Sampling of aerosol particles entering the recipient chambers showed similar particle size profiles for the two exposure chambers (**Figure 2**) except for the largest particles. The fraction of airborne particles >5 µm reaching the recipient chamber was 0% in the side-by-side exposure apparatus and 1% in the tunnel exposure apparatus. Total number of aerosolized particles per minute passing through the tunnel varied markedly during the typical 3-hour exposure, depending in part on the observed activity of the donor ferret (**Figure 3**).

### Exhaled Aerosol Transmission Required a Donor Threshold Viral Load

**Figure 4:**
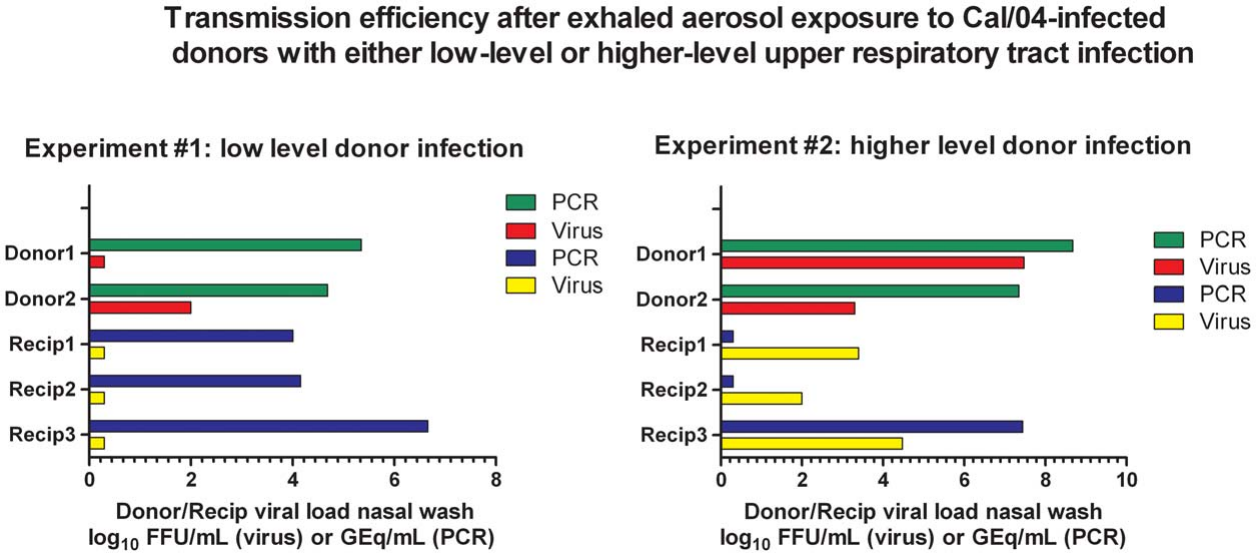
Exhaled aerosol transmission requires a donor threshold viral load. Two pairs (designated A and B) of donor ferrets (designated donor1 and donor2 of pair A, donor3 and donor4 of pair B) were infected i.n. and i.t. with a total dose of 10^6^ FFU of Cal/04. Three recipients (R1, R2, R3 exposed to pair A; R4, R5, R6 exposed to pair B) were exposed individually to one of the donor pairs for 3 h approximately 24 h after donor infection. Exposures to donor ferrets Donor1 and Donor2 with low viral loads in nasal wash (green and red, left graph) did not result in transmission indicated by negative cultures (yellow squares) in each of the 3 recipients, whereas exposure to ferrets with higher titers of nasal wash virus (red bar) resulted in transmission and positive nasal wash cultures (yellow bars, right graph). Viral RNA (‘PCR’) is expressed as genome equivalents/mL nasal wash; Viral culture (‘virus’) is expressed as FFU/mL nasal wash.

Pairs of donor ferrets were infected i.n. with 10^6^ FFU of Cal/04 and 3 recipients were exposed in the tunneled chambers to the donor pair for 3 h approximately 24 h after donor infection. Exposures to ferrets with low viral loads in nasal wash (**Figure 4**, left graph) did not result in transmission indicated by negative cultures in the recipients, whereas exposure to culture-positive ferrets resulted in transmission and positive nasal wash cultures (right graph). Positive cultures in the donors and recipients correlated with levels of viral RNA >10^7^ GEq in these experiments reported here and for a seasonal H1N1 virus [32]. Thus the level of mucosal viral load in the donor determined in part donor contagion under these experimental conditions.

### Comparison of Transmission after Exposures for 3 or 20 Hours

**Figure 5:**
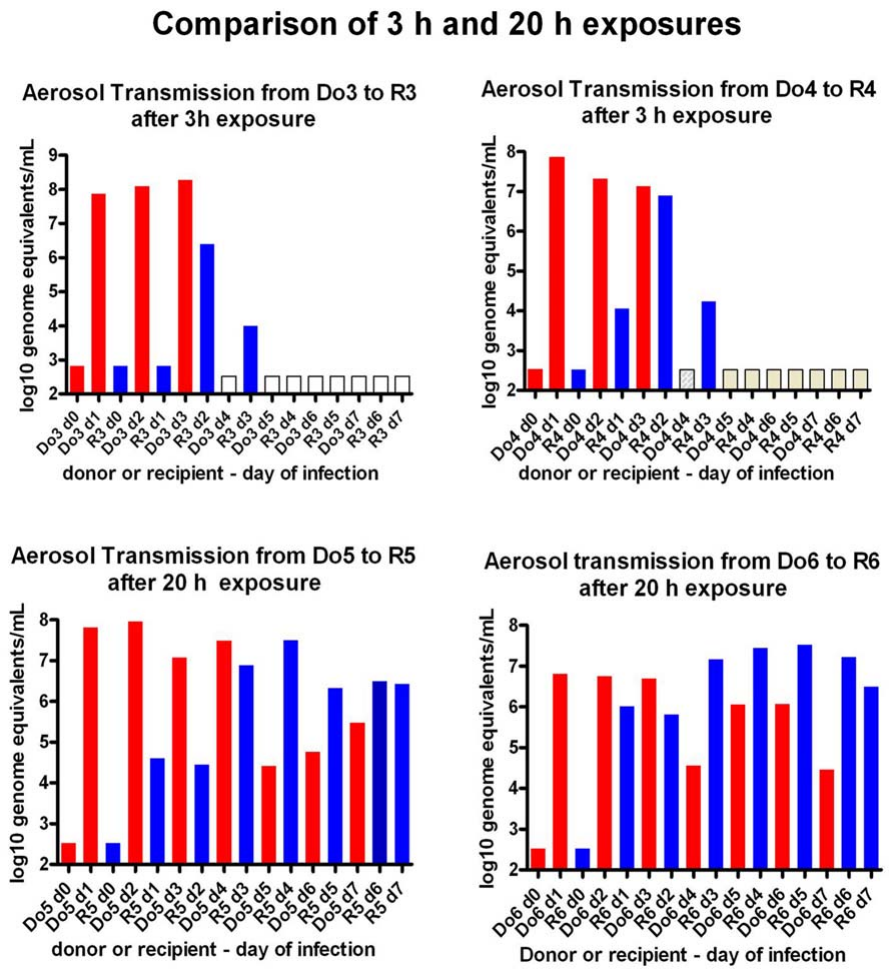
Aerosol transmission is more efficient after 20 h- than 3 h- exposures. Viral RNA (log_10_ genome equivalents (GEq) in total sample) in throat swabs from four donors (Do, red) infected intranasally 24 hours previously with Cal/04, and in four recipients (R, blue) each exposed to exhaled aerosols of one of the donors for either 3 hours (A and B) or 20 hours (C and D).

To compare previously published continuous exposures (>24h) in side-by-side cages [26]–[31] with 3 h exposures in similar cages, donor ferrets were infected i.n. with 10^6^ FFU of Cal/04, and one day later susceptible ferrets were exposed to donor exhaled viral aerosols. Infection was monitored by nasal washes twice and throat swabs daily for 5-days post-exposure (dpe) (**Figure 5** **)**. The two recipients exposed for 20 hours lost weight and had high levels of viral RNA for 5 days. In contrast, recipients exposed for only 3 hours had lower levels of viral RNA in the throat below the threshold of infection.

### Contagion Induced by Nebulized Virus

**Figure 6:**
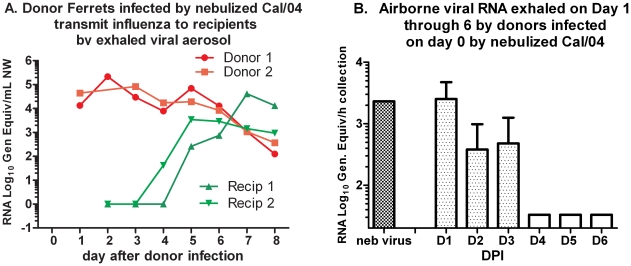
Aerosol Transmission from donors infected by aerosolized virus. Transmission of Cal/04 between donors infected by nebulized virus and recipients exposed continuously for 4 days. **A**. Serial viral RNA titers from throat swabs of donors and recipients, and samples of aerosols collected during continuous exposures, measured by single-target RT-qPCR. **B**. Viral RNA captured on PTFE filters during a 1 hour interval each day after donor ferret infection, and measured by T5000 assay.

Infection of donor ferrets by the intranasal route versus inhalation of nebulized virus could possibly induce differing efficiencies of contagion. Two donor ferrets were infected during a 1-hour exposure to nebulized Cal/04 virus, calculated to deliver a dose of 10^6^ FFU approximating the intranasal doses used above. In contrast to intranasal inoculation donors infected by nebulized virus had decreased activity 2−6 days post-inhalation (dpi), sneezing 2−7 dpi, diarrhea 2−8 dpi, elevated temperature >2.5°C above baseline at 5 dpi and loss of 6% and 11% of body weight, respectively. Each ferret exposed to one donor in the chamber continuously for four days had temperature elevations >2.0°C on dpi 3−7 and 5, respectively, and loss of 5.6% and 7.7% body weight, respectively, but without sneezing or diminished activity. Donors had high viral load in the throat at 1 dpi after nebulized virus exposure, whereas the recipient ferrets had high viral load delayed until 4 days post-exposure (dpe) after exhaled aerosol exposure (**Figure 6**A). Donor-exhaled viral RNA aerosol levels were highest at 1 dpi and were quantitatively similar to the nebulized viral RNA levels delivered to the donors during the 1-hour exposure at 0 dpi (**Figure 6B**). Thus, contagion induced by nebulized virus appeared to be at least as potent as that induced by intranasal inoculation, and continuous exposure resulted in symptomatic recipients. Additional head-to-head comparisons are needed, however, to identify any significant differences in infections transmitted by inhaled aerosol from nebulized virus inhalation versus liquid droplet inoculation.

### Aerosol Transmission During Early and Late Donor Infection

**Figure 7:**
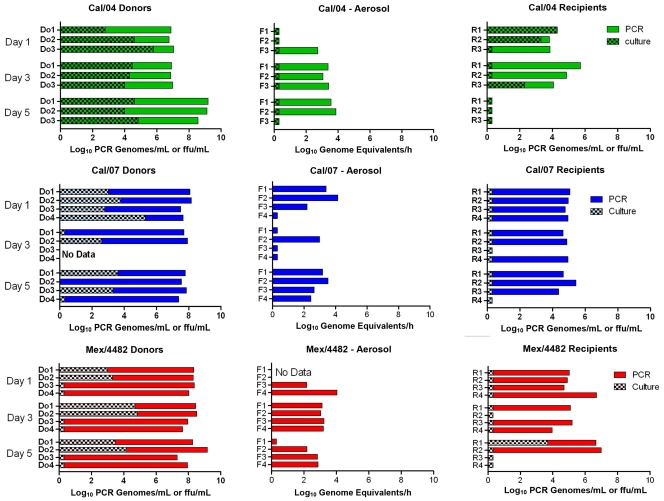
Comparison of Transmission efficiency of pandemic 2009 H1N1 strains during early and later phases of donor infection. Single donor-single recipient exposures in which the interval between donor instillation and recipient exposure was varied from 1, 3, or 5 donor dpi. Viral culture (hatched bar) is expressed as FFU/mL (total sample) of nasal wash collected on day of exposure (donors) or the greatest value found on day 1−3 post-exposure (recipients). Aerosol data are expressed as genome equivalents/1-hour filter collection; each of three filters was measured in triplicate, and the mean of the three filters reported. For each virus: Cal/04 (green), Cal/07 (blue) and Mex/4482 (red), the donor-recipient pair are aligned horizontally across the figure.

Observations in human infections have indicated that optimum contagion occurs during early infection prior to symptom appearance. To test for more efficient transmission in early infection, recipients were exposed to single donors 1, 3, or 5 days after the donors’ intranasal instillation. Donor viral loads in the upper respiratory tract were comparable among the three pandemic H1N1 strains by both culturable virus (approximately 10^4^ FFU/mL) and levels of viral RNA (6–9 log_10_ genome equivalents/mL) for most donors of each strain (**Figure 7**). The exhaled viral RNA levels from each donor also were not significantly different among the three strains. No correlation between level of airborne viral RNA and subsequent transmission could be drawn, however, as six exposures resulted in transmission in the absence of detectable viral RNA in the aerosol. One day and 3 days after donor instillation of Cal/04, all exposed recipients were infected by viral RNA criteria and half by culture-positive criteria. None of the 3 ferrets exposed to culture-positive day 5 Cal/04-infected donors were infected. When results from all three strains are combined, donors on their fifth day of infection were significantly less likely to infect recipients by the viral RNA criteria (5/11 vs 11/11 on dpi 1, *p* = 0.006, Fisher’s exact test). Transmission from Mex/4482- and Cal/07-infected donors was reduced 3 and 5 days after inoculation likely due to fewer culture-positive nasal washes or throat swabs in the donors.

### Comparison of Transmission Efficiency between 1 Seasonal and 3 Pandemic H1N1 Strains

**Figure 8:**
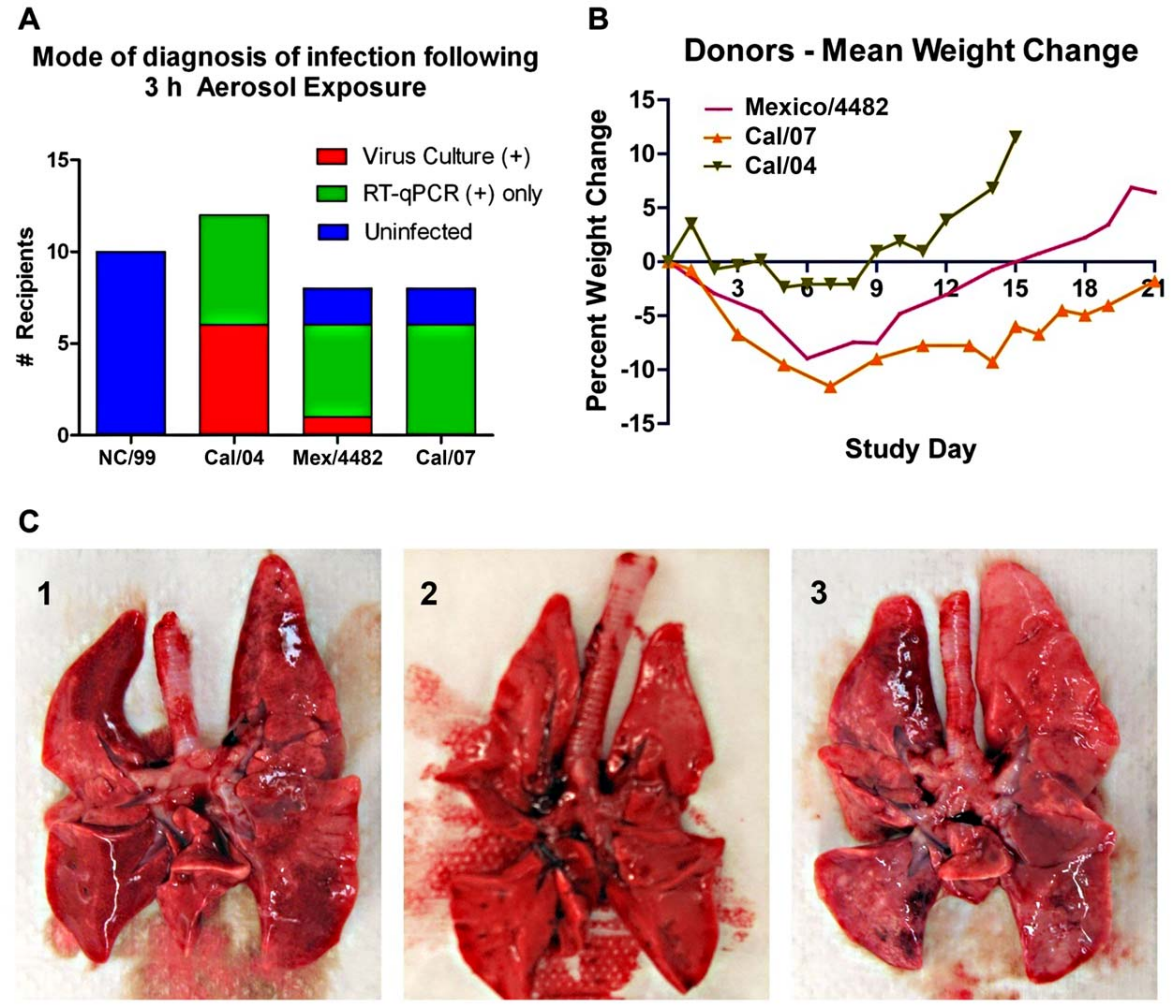
Efficiency of transmission success is inversely correlated with level of illness in the ferret aerosol-donor. A . Comparison of percent transmissions detected by positive culture or positive RT-qPCR of nasal washes or throat swabs of recipients tested 1−3 days post-exposure, for each strain of pandemic H1N1 2009 influenza A virus. Lack of transmission of NC/99 is shown as left-hand column for comparison. **B**. Group mean weight change in the donors following intranasal infection with the three pandemic H1N1 strains. **C**. Photographs of whole lung tissue at necropsy (day 5) of donor ferrets infected with Cal/04 (C2, middle photo) or Cal/07 (C1 and C3). Cal/07-infected lungs display multiple regions of firm, dusky tissue representing pneumonitis confirmed by histology, not seen in Cal/04-infected lungs.

Nebulized seasonal virus NC/99 was infectious to ferrets with a low aerosol ID_50_, although culturable virus was rarely present in the upper airways [32]. To test whether this strain could be transmitted by exhaled aerosol, donor ferrets were infected i.n. with 10^6^ FFU of NC/99, or one of the three pandemic 2009 H1N1 strains as described above, and susceptible ferrets were exposed to their exhaled aerosols 1 day later (summarized in **Figure 8A**). Using the criterion of viral load either by culture or by viral RNA, there was no evidence that NC/99 was transmitted in any of 10 exposures using either a pair of infected donors (double dose) or a single infected donor. In contrast, all 12 exposures to single Cal/04-infected ferrets resulted in infections, half detected by culture and the remainder by RT-qPCR and the T5000 assay. Almost all of the ferrets infected with the other two 2009 H1N1 pandemic strains had evidence of infection by viral RNA in the throat and nasal wash, but only 1 of 16 had positive cultures, as noted in Figure 7. No Cal/07-exposed ferrets developed culture-positive infections, significantly fewer than those in Cal/04 infections (Fisher exact test, *p* = 0.006).

Intranasal inoculation of the donor ferrets induced varying degrees of illness depending on the pandemic H1N1 strain. Donors infected with Cal/04 transmitted more culture-positive infections, yet exhibited the least weight loss (**Figure 8B**), no gross lung pathology (**Figure 8C**) and the fewest respiratory symptoms (**Table 1**). In contrast, Cal/07 donors experienced the most weight loss, the most apparent pulmonary inflammation at necropsy (**Figure 8C**) and the most respiratory symptoms (**Table 1**), yet failed to transmit culture-positive infection (**Figure 7**). The Mex/4482 virus was intermediate between the two other strains in these measures of illness. This apparent inverse relationship between less disease and more efficient transmission requires more comparative investigations to extend these observations, but suggests an effect of the inflammatory response on aerosol transmission.

**Table 1:** Symptoms exhibited by donor ferrets following intranasal infection with one of three pandemic H1N1 strains.

Donors Sign Of Onset	NC/99 (N = 4)		Cal/04 (N = 25)		Mex/4482 (N = 12)		Cal/07 (N = 10)	
	Median^a^ (Range)	% Sx^b^	Median (Range)	% Sx	Median (Range)	% Sx	Median (Range)	% Sx
Max temp Δ °C	0.4 (0.6–0)	-	0.6 (0–0.8)	-	1.0 (0.2–1.4)	-	1.3 (0.6–2.1)	-
Activity decrease	0	0	0.1	8	0.1	8	0.8	80
Nasal Discharge	-	-	D1 (D1–5)	24	D2 (D1–20)	58	D1 (D-4–18)	100
Neurological	-	-	-	-	-	-	D8 (D8–9)	20
Ocular Discharge	-	-	-	-	-	-	D3 (D2–11)	40
Sneezing	-	-	-	-	D8 (D1–19)	50	D2 (D2–9)	50
Stool	-	-	D1 (D1)	12	D2 (D1–19)	41	D2 (D2–11)	80

a group median for change in temperature or day (D) of onset of symptom and range of days post-infection that symptoms were observed.

b Percent of group with activity decrease or symptom.

### Relationship of Exhaled Viral RNA Levels to Transmission

**Figure 9:**
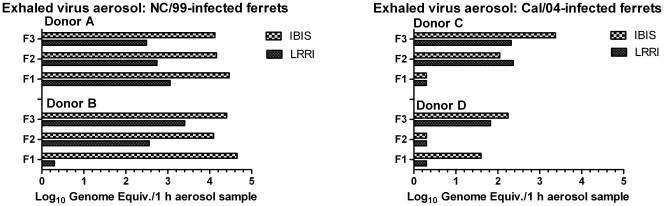
Exhaled viral RNA from donors infected with either NC/99 (A and B) or Cal/04 (C and D) virus. RNA was measured in each filter sample by two RT-qPCR-based assays, detecting a single genomic RNA segment (LRRI assay, solid bar) or six segments (Ibis T5000 assay, checkered bar), and expressed as genome equivalents/1-hour filter collection. Donors were infected intranasally with 10^6^ FFU of either virus 24 hours prior to recipient exposure at a time when all donor nasal washes contained 10^4^–10^5^ FFU/mL. Each donor exposed three recipient ferrets and each of their corresponding collections (F1, F2, and F3) were the mean RNA levels from three filters each collecting airborne particles for 1 hour during the 3-hour exposure period.

A higher viral load in upper airway mucosa might be expected to result in more efficient aerosol transmission. Higher donor viral load in Cal/04 infection was associated with transmission in Figure 4, and in all experiments donor ferrets with less than 3 log_10_ FFU/nasal wash cultures never transmitted culture-positive infection, consistent with a donor threshold. On the other hand, donors with high levels of culturable pandemic H1N1 virus in nasal wash failed to transmit infection. Levels of virus in exhaled aerosols might be a more direct measure of contagion but measured viral RNA in exhaled aerosols was not clearly related to transmission of the pandemic H1N1 strains (**Figure 7**). Levels of viral RNA in exhaled aerosols were compared between the nasal-wash culture-positive NC/99-infected donors and the NW culture-positive Cal/04-infected donors. Under identical exposure conditions the NC/99 virus was not transmitted, compared to frequent transmission of Cal/04, yet NC/99-infected donors exhaled significantly higher levels of viral RNA measured by either assay than Cal04-infected donors (**Figure 9**). Transmission of Cal/04 occurred in spite of viral RNA-negative 1 h filter collections, likely attributable to highly variable levels of exhaled virus during the 3 h exposure.

### Source of Exhaled Virus

**Figure 10:**
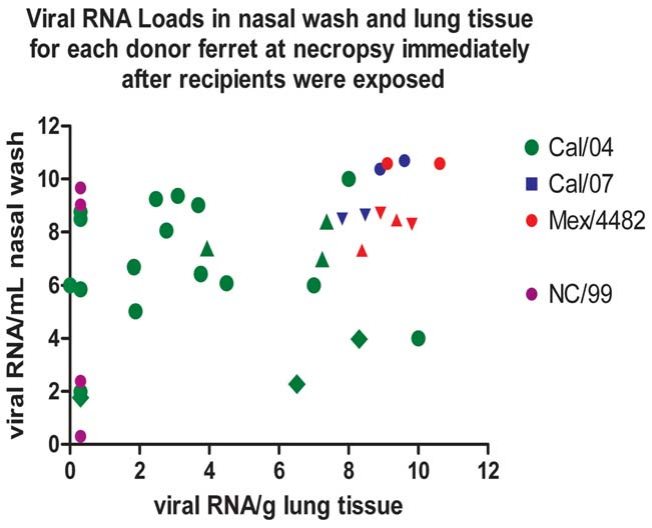
Viral loads (RNA genome equivalents per mL nasal wash or per g lung tissue) for each donor ferret on dpi 1 or dpi 3. Necropsy of donor was performed 2 h after exposing recipient ferrets. Data combined from 3 experiments indicated by circles, triangles and diamonds. Lung tissue viral RNA levels for Cal/04 were mean (SD) = 5.94 (2.72) compared to Cal/07+Mex/4482 lung RNA mean (SD) = 8.84 (1.42), significant by t test at p = 0.007. Aerosol transmission success for each virus is exhibited in Figures 4 and 7. The NC/99-infected donors did not have viral RNA in lung tissue nor transmitted infection by exhaled aerosol.

Exhaled virus from contagious ferrets may be derived from both upper airway mucosa and small airways within the lung, and thus upper airway cultures may not reflect the total source of the exhaled viral aerosol. Viral load for each of the four influenza viruses was graphed according to both the upper airway viral RNA load and viral RNA levels in the lung of donor ferrets assessed on the day of exposure (**Figure 10**). Cal/07- and Mex/4482-infected ferrets had significantly higher viral loads measured by viral RNA levels in lung tissue than Cal/04-infected ferrets (*p* = 0.007), but these higher viral loads were associated with lower rates of transmission. The NC/99-infected donors did not have viral RNA in lung tissue nor transmitted infection by exhaled aerosol. Thus higher lung viral RNA titers were not associated with higher aerosol transmission efficiency among pandemic H1N1 strains, but a threshold level of virus required for transmission was not established. Imaginative experimental designs will be needed to determine the tissue source of exhaled virus.

### Airway Culture-negative Lung Infection in Aerosol-exposed Ferrets

**Figure 11:**
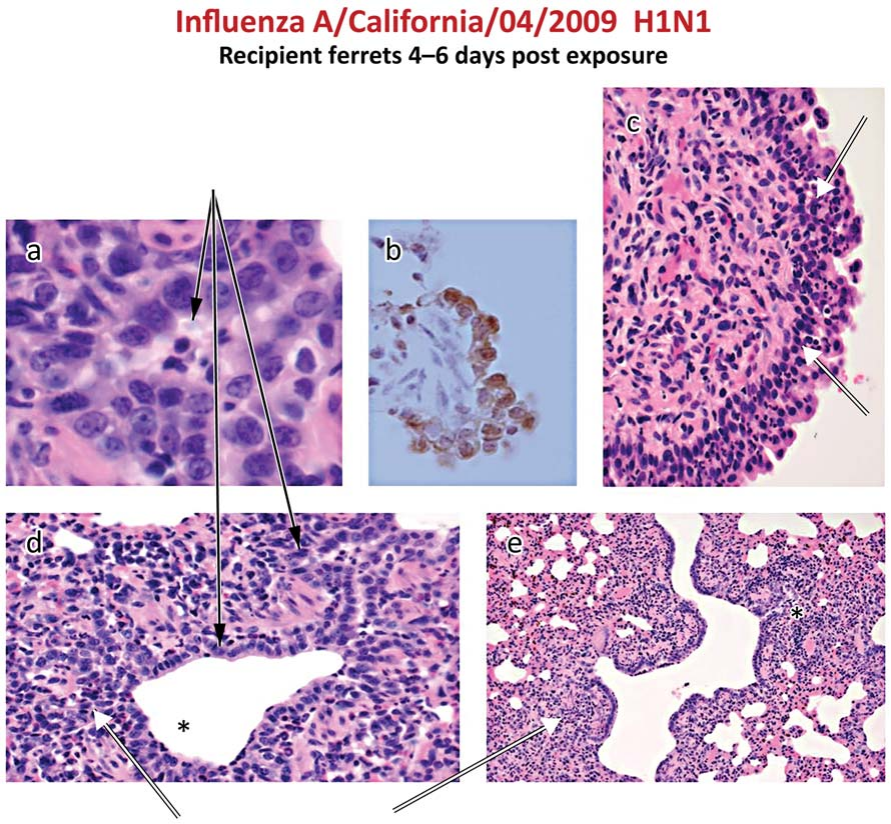
Histology of lung and turbinates from exhaled aerosol-exposed recipient ferrets infected with Cal/04. a,d: Mild focal bronchiolitis with epithelial cell karyomegaly and desquamated epithelium and neutrophil inflammatory cells in the lumen of small airways (solid arrows). b. Immunohistochemical detection of influenza nucleoprotein antigen in airway epithelial cells. **c,d,e.** Intramucosal and submucosal inflammatory cells in the turbinate (c) and peribronchial tissue (d,e).

Detection of culture-negative viral RNA in the recipient ferret airways raised the possibility of either a low level of infection that was not contagious or that no infection had been established. Recipient ferrets were necropsied at intervals after exposure for histological analysis (**Table 2**) and a sample of 15 lungs containing typical histologic characteristics of influenza infection were stained for viral antigen by immunohistochemistry (IHC). All ferrets with viral RNA in lung tissue after exposure to the pandemic H1N1 viruses had evidence of infection although it was graded as either minimal or mild in most cases. No recipient exposed to the NC/99 virus had histologic evidence of infection, consistent with the absence of viral RNA in the lung (**Figure 10**). Histological evidence of infection included epithelial karyomegaly and necrosis in terminal bronchioles with peribronchiolitis, and acute inflammation in the nasal turbinates (**Figure 11** **A, C, D, E**).

**Table 2:** Histologic grades of inflammation in respiratory tract tissues of ferrets infected with three pandemic H1N1 viruses.

Histologic grades of inflammation in nasal turbinates and lung tissue on day 5 or 6 post exposure to exhaled aerosol from donors infected with virus 24 h prior to exposure.	Cal/04	Cal/07	Mex/4482
Number of recipient ferrets with histological alterations in the respiratory tract (%)	14/15	3/4	4/4
	93%	75%	100%
Nasal Cavity Inflammation, acute	14	3	4
Minimal	14	3	3
Moderate	0	0	1
Lung Bronchiole Inflammation, acute	9	1	3
Minimal	4	1	2
Mild	2	0	1
Moderate	3	0	0
Pneumonitis, interstitial^b^	12	2	3
Minimal	5	2	2
Mild	7	0	1

Among 9 recipient lungs infected with Cal/04 according to viral RNA criteria alone, 7 were positive by IHC (**Figure 11B**). Of 14 recipient lungs that were viral RNA negative, 9 were IHC-negative, but 5 were IF-positive. This discrepancy suggests that in low-level infections the extent of tissue sampling will impact interpretation. Thus aerosol exposures may result in culture-negative lung and turbinate infections manifest only by viral RNA and viral antigen to indicate established but weak viral replication.

### Calculation of the Aerosol Infectious Dose of Pandemic H1N1 Influenza A

**Figure 12:**
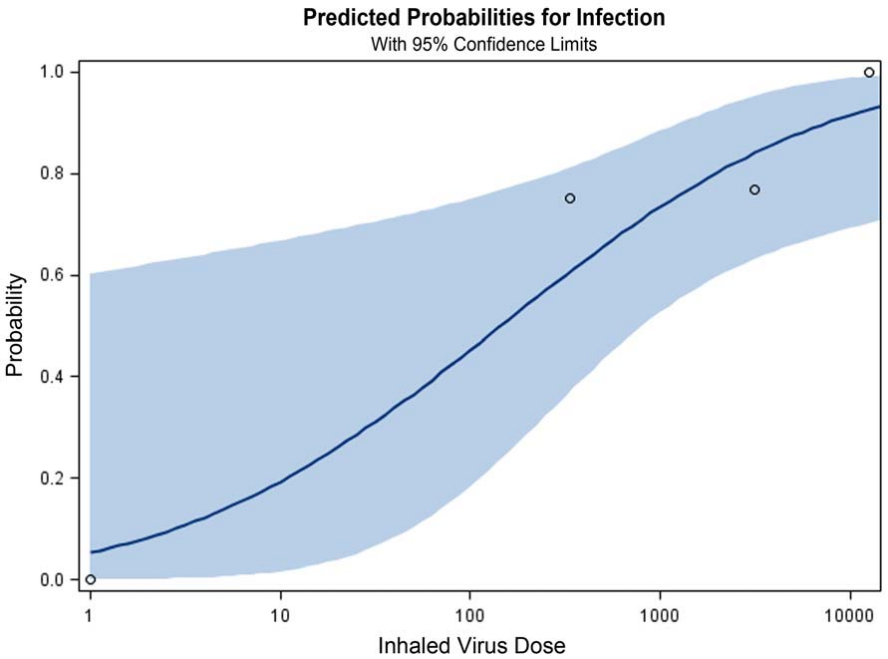
Calculation of the infectious dose for exhaled aerosol Cal04. The Proc Probit procedure was used to calculate the infectious dose causing 50 percent of ferrets to be infected, as log_10_ dose = 2.16 (145 genome equivalents). The 95% confidence levels are –1.69 and 2.913 (0.02 and 819 genome equivalents).

To calculate the aerosol ID_50_ of Cal/04 for recipients on days 1 and 3 post-exposure, the data were grouped according to the following criteria: For log_10_ dose <1 is group one, 2<log_10_ dose< = 3 is group two, 3<log_10_ dose<4 is group three, log_10_ dose >4 is the group four. For each group, the mean is used as the log_10_ dose for each group. The Proc Probit procedure in SAS (version 9.0 Cary NC) was used to calculate the infectious dose causing 50 percent of animals to be infected. The log_10_ dose causing the 50 percent of animals infected is 2.16 (145 genome equivalents). The 95% confidence levels are -1.69 and 2.913 (0.02 and 819 genome equivalents) (**Figure 12**). Using the aerosol viral RNA levels and the viral RNA:viable virus ratio developed previously [32], the aID50 was estimated at 1 virus particle (95% confidence limits: 0.1–3.0).

## Discussion

Our calculation of the minimal infectious dose of exhaled airborne Cal/04 pandemic virus as approximately 1 FFU is consistent with studies in ferrets and humans using nebulized virus. Using the same exposure chambers in this study, we reported the aerosol ID_50_ of the H1N1 NC/99 seasonal virus to be 4 pfu [32], although this NC/99 transmitted infection was detected only by seroconversion and viral RNA, and not by symptoms or viral culture. The minimal nebulized H3N2 seasonal virus dose inducing symptomatic infection in ferrets was 2 pfu by the aerosol route [31] compared to 1 pfu by the intranasal route. The minimal aerosol dose by nose-only exposure in ferrets was 1 pfu for a virulent avian H5N1 strain [37]. Intranasal minimal infectious dose of an H5N1 strain in mice was 4 pfu [38], and an H3N2 strain in guinea pigs was 5 pfu [39]. Human volunteers exposed to a nebulized seasonal H3N2 virus developed symptomatic infection from a calculated mean infectious aerosol dose of 0.3 to 6 TCID_50_ [40], in contrast to substantially higher minimal doses by intranasal drops [41]. Although aerosol ID_50_ measurements for nebulized versus exhaled virus may not be exactly comparable, infectious doses transmitted by aerosol appear to be very low. If influenza transmission among ferrets is comparable to inter-human transmission, low aerosol infectious doses may have implications for designing public health interventions.

Measuring aerosol transmissibility in animal models is dependent on the limitations imposed by the experimental conditions. Brief whole-body exposure between unrestrained, non-anaethetized ferrets was chosen for this study to approximate the conditions of transmission among humans in their daily activities. The advantages of this design include natural respiration patterns, expected dilution of airborne virus by deposition on the inanimate environment, and exposure to low concentrations of virus in the aerosol. The disadvantages include inability to exclude exposure by the non-inhalation routes, loss of aerosolized virus on the surfaces of the origin chamber, and the need for more surface decontamination between exposures. These disadvantages are advantages of the nose-only exposure systems [37], [42], but the reduced minute ventilation of the ferret sleeping in the conical restraint may alter the mucosal distribution of inhaled virus. Airborne virus has a low death rate constant if the humidity is low [43], experimentally confirmed in the guinea pig model by highest transmission at 20% relative humidity [39], [44]–[46]. Exposures in this study were conducted at relative humidity of approximately 45%, possibly reducing the efficiency of transmission.

Our chamber designs intentionally limited the exposure to small-particle exhaled virus, less than 10 µm in the tunneled chamber and less than 5 µm in the un-tunneled chamber. We observed no marked difference in transmission efficiency between the two chambers. In a study of ferrets exposed to exhaled H3N2 virus with a size profile similar to ours [31], the cascade impactor captured more of the airborne infectious particles in the 4.7 µm diameter cohort in the two ferrets analyzed. It is possible that our chambers excluded significant numbers of infectious particles from reaching the susceptible ferrets, thus reducing culture-positive infections. Humans exhale droplets of widely varying sizes and quantity during normal breathing and talking [7], [47]. Exhaled infectious particle size needs further investigation, since virus in aerosol particles less than 3 µm diameter remain suspended in the air for an hour or more thus increasing opportunities for aerosol transmission.

Quantifying aerosol transmissibility in animal models is dependent on the sensitivity of assays detecting airborne virus. The PTFE filter used in this study has been shown to be more efficient than the AGI impinger in capturing viral RNA but less efficient in capturing infectious particles [48]. During short collection intervals of 10 min or less both filters [32] and liquid impingers [31], [32] are efficient collectors. However, the marked variability of exhalation of particles, and presumably virus, over time is apparent in our data (figures 3, 7, 9) and in others (figure 1, [31]), suggesting that short periods of collection may not be reliable to capture total exhaled virus levels. In this study PTFE filter collections for 1 h were rarely culture-positive for virus and only for the Cal/04 virus, so we elected to use viral RNA collected during the entire exposure to estimate airborne virus inhalation. Influenza RNA, but not cultured virus, was detected in aerosols exhaled from three of five individuals infected with influenza A [49]. In cough specimens from subjects with acute influenza A, 81% had viral RNA detected by M segment RT-qPCR but only 2 of 21 had cultured virus [47]. We derived the ratio of total viral RNA to infectious particles from filters exposed to nebulized virus for only 20 minutes [32]. The lower ratio calculated using cascade impactors [32] may be due to destruction of intact genomic RNA [48]. Ratios of RNA genome equivalents to infectious virions have been subject to wide experimental variation. Viral particles secreted into tissue culture media consists primarily of non-infectious (defective interfering) particles among only 1% infectious particles [50]–[52]. Future work must address these technical limitations of quantifying low levels of aerosol infectious particles.

Continuous exposures to exhaled aerosols with seasonal and pandemic strains have resulted in culture-positive infections and seroconversions [23]–[30]. In this study, however, neither symptoms nor culture-positive airways were commonly documented. Seroconversion was not used in this study because lung histopathology and viral load was examined during the first week after exposures. Many exposed ferrets with negative airway cultures had viral RNA-positive airways for 5 days after exposure. This observation was interpreted to indicate established mucosal infection, since UV-inactivated viral RNA deposited on ferret mucosa is cleared and undetectable within 24h [32]. We documented established lung infection with the pandemic H1N1, but not seasonal H1N1, viruses by immunohistochemical detection of viral antigen in culture-negative, viral RNA-positive ferrets. Nonetheless the culture-negative infections likely indicate that the viruses studied were significantly less virulent than that reported for the H3N2 strain [31]. Whether 3 h exposures to less virulent strains are contagious and provide immunologically relevant exposure should be tested.

We compared three closely related pandemic 2009 H1N1 viruses for aerosol transmissibility and virulence. Intranasal infection with Cal/07 and Mex/4482 clearly induced greater morbidity, fever, weight loss, sneezing, and decreased activity, which were all absent in the ferrets infected with Cal/04. The greater morbidity of Cal/07 has been observed previously [53] as has the mild morbidity in ferret Cal/04 infections [29]. The genomic basis for this virulence heterogeneity is unknown, as only four nucleotide differences among the eight RNA segments were found between the Cal/04 and Cal/07 stocks we used (P Gao, unpublished data). Single nucleotide mutations in the hemagglutinin, neuraminidase, and PB2 segments have been shown to alter transmissibility [21], [23]–[27], but these mutations were not present in the strain isolates studied here.

The two more virulent strains were less efficiently transmitted using the criteria of few culture-positive recipients, yet upper airway donor viral loads were similar among the three pandemic viruses. Moreover the levels of viral RNA in exhaled aerosols were also comparable. We speculate that either the donor’s mucosal inflammatory response diminished exhalation of viable virus, not distinguished by a viral RNA assay, or that aerosol-derived infection was a function of the relative replication efficiency of virulent virus strains in susceptible respiratory mucosa. In a recent study [54] we used computational modeling of viral kinetics in human tracheal epithelial cells to derive estimates of strain-specific viral productivity per infected cell and the spread of virus from a primary to secondarily infected cells (basic reproductive number R_0_). The replication efficiency of the pandemic Cal/04 strain was 5−10-fold greater than the seasonal NC/99 strain [54]. Similar differences between replication rates have been documented in ferret tracheal epithelial cells (unpublished data). Thus, successful transmission from contagious to susceptible ferret may depend as much on the replication efficiency of the virus strain as on the inhaled dose of viable virus and other virus-host relationships.
